# The regulation of skin homeostasis, repair and the pathogenesis of skin diseases by spatiotemporal activation of epidermal mTOR signaling

**DOI:** 10.3389/fcell.2022.950973

**Published:** 2022-07-22

**Authors:** Juan Wang, Baiping Cui, Zhongjian Chen, Xiaolei Ding

**Affiliations:** ^1^ Institute of Geriatrics (Shanghai University), Affiliated Nantong Hospital of Shanghai University (The Sixth People’s Hospital of Nantong), School of Medicine, Shanghai University, Nantong, China; ^2^ Shanghai Engineering Research Center of Organ Repair, School of Medicine, Shanghai University, Shanghai, China; ^3^ School of Medicine, Shanghai University, Shanghai, China; ^4^ Shanghai Engineering Research Center for External Chinese Medicine, Shanghai, China; ^5^ Shanghai Skin Disease Hospital, School of Medicine, Tongji University, Shanghai, China

**Keywords:** mTOR, epidermis, keratinocytes, wound healing, metabolism

## Abstract

The epidermis, the outmost layer of the skin, is a stratified squamous epithelium that protects the body from the external world. The epidermis and its appendages need constantly renew themselves and replace the damaged tissues caused by environmental assaults. The mechanistic target of rapamycin (mTOR) signaling is a central controller of cell growth and metabolism that plays a critical role in development, homeostasis and diseases. Recent findings suggest that mTOR signaling is activated in a spatiotemporal and context-dependent manner in the epidermis, coordinating diverse skin homeostatic processes. Dysregulation of mTOR signaling underlies the pathogenesis of skin diseases, including psoriasis and skin cancer. In this review, we discuss the role of epidermal mTOR signaling activity and function in skin, with a focus on skin barrier formation, hair regeneration, wound repair, as well as skin pathological disorders. We propose that fine-tuned control of mTOR signaling is essential for epidermal structural and functional integrity.

## Introduction

**FIGURE1:**
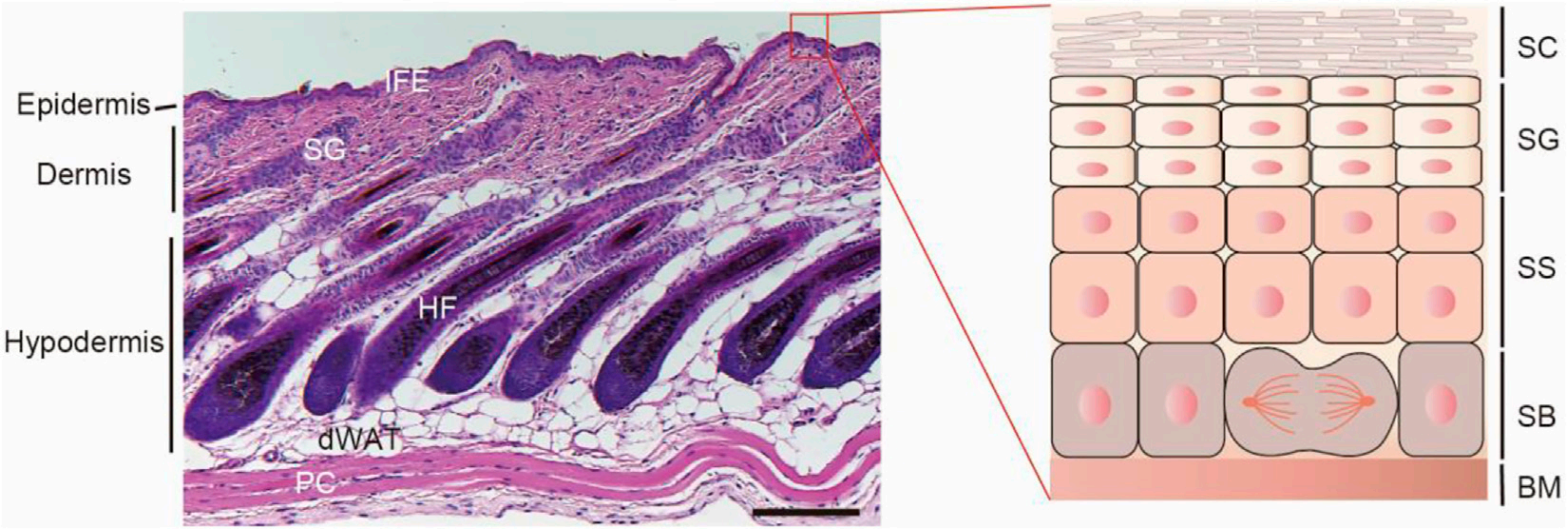
The skin architecture. Left, hematoxylin and eosin (H&E)-stained P32 mouse back-skin section. The skin is composed of three layers: epidermis, dermis and hypodermis. The epidermis is the out layer and compartmentalized into interfollicular epidermis and its appendages, such as hair follicle and sebaceous gland. IFE, interfollicular epidermis; SG, sebaceous gland; HF, hair follicle; dWAT, dermal white adipose tissue; PC, panniculus carnosus muscle. Scale bar, 100 µm. Right, scheme of interfollicular epidermal structure. According to the keratinocyte differentiation stage, cell morphology and marker expression, IFE can be distinguished into four layers: stratum basale (SB), stratum spinosum (SS), stratum granulosum (SG), and the stratum corneum (SC). SB is attached to the basement membrane (BM), which separates the epidermis and dermis.

Mammalian epidermis, the outer layer of the skin forms a vital barrier between the body and the external world. The epidermis is comprised of the interfollicular epidermis (IFE) and the interspersed pilosebaceous units, such as the hair follicle (HF) and sebaceous gland (Fuchs, 2007). Each compartment undergoes constantly self-renewal or a perpetual cycle of growth during postnatal homeostatic and regenerative processes. The inner basal layer cells of IFE are mainly stem cells or proliferative progenitors. After detaching from the basement membrane (BM), they migrate upwards and undergo through a tightly organized differentiation process to yield the outer layers, including stratum spinosum (SS) layer, stratum granulosum (SG) layer, and the stratum corneum (SC) (Figure 1). SC is the outmost layer, consists of dead and keratin-filled corneocytes embedded in a lipid matrix and confers the major barrier function of the skin (Eckhart et al., 2013). Under physiological condition, SC are continually shed from the tissue and subsequently replaced from the other differentiating layer cells (Candi et al., 2005). Additionally, the skin is frequently challenged by various external stress factors, which results in tissue injury and barrier damage. As such, the epidermis needs constantly to restore damaged structure to re-establish the barrier function (Eming et al., 2014). Thus, far from being a static- physical barrier, the epidermis is a highly dynamic tissue, reacting to environmental stimuli, replacing cells and repairing the damaged region to maintain the skin barrier integrity. Perturbation of epidermal barrier integrity makes the skin vulnerable to the outside allergens, irritants and microbial infections, leading to the development of a variety of inflammatory skin diseases, such as atopic dermatitis (AD), psoriasis, and even oncogenesis (Segre, 2006; Kubo et al., 2012; Darido et al., 2016).

Keratinocytes, the major cell type in the epidermis need engage in an active metabolism rewiring to fuel their growth, proliferation, migration, and differentiation. It is therefore of particular importance to characterize changes in metabolic pathways during epidermal homeostasis (Cibrian et al., 2020). Mechanistic target of rapamycin (mTOR) signaling is a central hub of cell growth and metabolism by sensing a myriad of environmental stimuli such as nutrients and growth factors (Saxton and Sabatini, 2017). It is not surprising that recent findings highlight that mTOR signaling is implicated in multiple aspects of epidermal homeostatic processes, including barrier formation, hair growth, and skin repair. More importantly, mTOR signaling activation appears to be temporal and regionally compartmentalized manners in the epidermis, depending on specific context and environmental cues (Squarize et al., 2010; Ding et al., 2016; Ding et al., 2020). Dysregulated mTOR signaling is associated with the development of various skin disorders, such as impaired wound healing (Goren et al., 2009), psoriasis, and skin cancer (Chen S. J. et al., 2009b; Buerger et al., 2013). In this review, we summarize the major recent findings on mTOR signaling, with a focus on the activation and function of mTOR signaling in skin epidermis. We envision that untangling these functional implications of mTOR activation during epidermal homeostasis will be critical for developing novel therapies for skin disorders.

## Overview of mechanistic target of rapamycin signaling pathway

**FIGURE 2:**
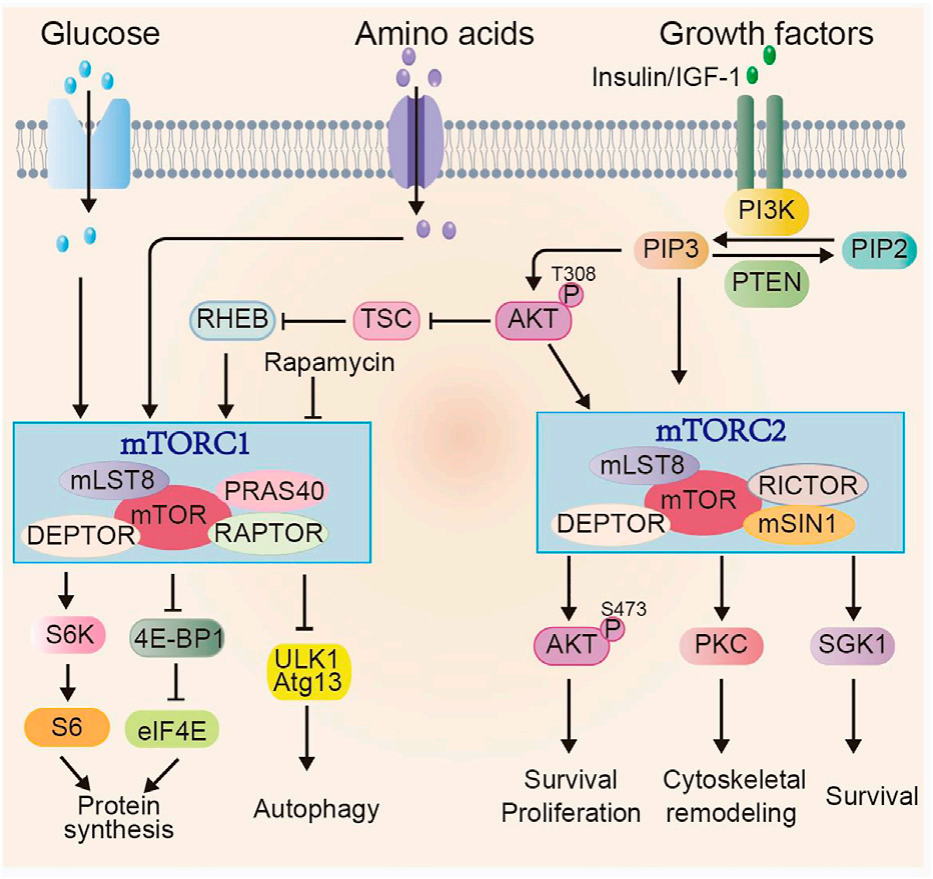
Overview of mTOR signaling pathway. mTOR exists in two complexes mTORC1 and mTORC2. Multiple factors, including glucose, amino acids and growth factors can induce mTORC1 activity through various signaling cascades. Activated mTORC1 promotes anabolic processes, e.g., protein synthesis through phosphorylating its downstream effectors, such as S6K and 4E-BP1 and inhibits catabolic process, such as autophagy. The regulation of mTORC2 activity is mainly through growth factors and PI3K signaling. The activated mTORC2 signaling regulates cell survival, proliferation and cytoskeletal re-organization through phosphorylating members of AGC family, including AKT (at Ser473), PKC and SGK1.

The mTOR signaling is highly conserved among eukaryotic species. mTOR protein is a serine/threonine kinase and exists in two complexes with distinct structure and function, referred to as mTOR complex 1 (mTORC1) and mTORC2 (Dobrenel et al., 2016; Saxton and Sabatini, 2017). Besides the catalytic subunit mTOR, mTORC1 contains several other components, including mammalian lethal with sec-13 protein 8 (mLST8), domain-containing mTOR-interacting protein (DEPTOR), the proline-rich Akt substrate 40 kDa (PRAS40), and the regulatory-associated protein of mTOR (RAPTOR). The regulation of mTORC1 mainly depends on the abundance of nutrients and growth factors. Growth factors, for instance, insulin and insulin-like growth factor 1 (IGF-1) induce PI3K-AKT signaling. PI3K-AKT further activates mTORC1 *via* phosphorylating the tuberous sclerosis complex (TSC) and also PRAS40, which are negative regulators of mTORC1. Activated mTORC1 enhances anabolic processes, such as protein, lipid and nucleotide synthesis, ribosome biogenesis, which subsequently promote cell growth and proliferation. mTORC1 can also inhibit catabolic processes such as autophagy (Saxton and Sabatini, 2017). For protein synthesis, mTORC1 can directly phosphorylate ribosomal protein S6 kinase (S6K), which further phosphorylates ribosomal protein S6. mTORC1 can also phosphorylate eIF4E binding protein 1 (4E-BP1). Phosphorylated 4E-BP1 release eIF4E, which is a mRNA cap-binding protein and regulates translation initiation. Cell autophagy is mediated by unc-51-like autophagy-activating kinase 1 (ULK1), a serine/threonine kinase, interacting with several proteins, such as ATG13. Activated mTORC1 directly phosphorylates a number of autophagy-associated proteins, such as ULK1, ATG13, suppressing their activities and the autophagy initiation and progression. Whereas when mTORC1 is inhibited, ULK1 kinase activity is enhanced, promoting autophagy (Deleyto-Seldas and Efeyan, 2021) (Figure 2).

In addition to the shared components (mTOR, mLST8 and DEPTOR) with mTORC1, mTORC2 contains its unique core subunits: rapamycin-insensitive companion of mTOR (RICTOR) and mammalian strepss-activated Map kinase-interacting 1 (mSIN1). mTORC2 is mainly activated by growth factors and can only be inhibited with prolonged rapamycin treatment (Fu and Hall, 2020). Activated mTORC2 phosphorylates AGC family including PKC, SGK1 and AKT, affecting cell survival, proliferation, and cytoskeletal remodeling (García-Martínez and Alessi, 2008; Yang et al., 2015; Baffi et al., 2021). Phosphorylated AKT at Ser473, a proved indicator of mTORC2 activity, further stimulates cell growth and proliferation *via* phosphorylating downstream effectors such as Forkhead box O1/3 (FOXO1/3a) (Masui et al., 2013).

Over the past decades, the role of mTOR signaling in aging, metabolism and cancer has been extensively studied (Cornu et al., 2013). It is well established that reducing mTOR signaling activity genetically or by pharmaceutical intervention can extend lifespan with diverse model organisms, including yeast, worms, flies, and rodents, improve many aspects of health and delay or prevent ageing-related diseases (Hansen et al., 2007; Niccoli and Partridge, 2012; Johnson et al., 2013; Saxton and Sabatini, 2017). However, application of rapamycin and its derivatives, also known as “rapalogs” to reduce mTOR signaling activity can also impair specific aspects of health, mostly notably impaired wound repair, which constitutes a major risk factor for chronic wound diseases (Squarize et al., 2010; Iglesias-Bartolome et al., 2012; Mori et al., 2014). mTOR signaling exhibits significant complexity and variety depending on the context and environmental cues (Cornu et al., 2013). It is therefore important to identify the timeframes of mTOR signaling activation during tissue homeostasis that allows to improve the health benefits and alleviate impairments through mTOR activity manipulation.

## Mechanistic target of rapamycin signaling is essential for epidermal barrier formation

During embryonic development, the epidermis originates from embryonic surface of ectoderm, consisting of a single-layered epithelium and flat cells (Koster and Roop, 2007). Epidermal barrier formation is a rapid, highly dynamic process, which is coordinated by multiple regulators and signaling pathways (Koster and Roop, 2007). In mice, the epithelial cells start to commit to differentiation at around embryonic day 9.5 (E9.5) and subsequently undergo both symmetric cell divisions to increase surface area and asymmetric cell divisions to enhance tissue thickness. The formation of SC permeability barrier takes place at around E16.5 from dossal to ventral sides and a fully functional barrier formation nearly completes at E18.5 (Hardman et al., 1998; Fuchs, 2007). The molecular signals that govern epidermal stratification are diverse. Using immunohistochemical staining, we observed a strong stained signal of phosphorylated mTOR and its downstream molecule 4E-BP1 throughout all the layers of E15.5 epidermis, whereas a robust signal for phosphorylated S6 is predominantly detected in the suprabasal layers starting at E16.5 (Ding et al., 2016), indicating that mTORC1 signaling is induced during epidermal stratification and differentiation. The crucial role of mTORC1 signaling activation to epidermal stratification and barrier formation is supported by the findings of several loss-of-function mouse models. Mice with epidermis-specific *mTOR* deficiencies die neonatally due to damaged epidermal stratification and severe skin barrier defects (Ding et al., 2016). Notably, epidermis-specific ablation of the mTORC1 components, *Raptor* or *Rheb1* largely recapitulate the phenotypes observed with epidermal-specific mTOR deficient mice (Ding et al., 2016; Asrani et al., 2017), suggesting that mTORC1 signaling is essential for epidermal morphogenic process and barrier formation.

Concerning mTORC2, it has been shown that its downstream effector AKT plays a variety of roles in the epidermal keratinocytes, ranging from the initiation of the differentiation program to the proper development of the cornified envelope during late terminal differentiation with cultured keratinocytes and *in vivo* studies (Calautti et al., 2005; O'Shaughnessy et al., 2007). Interestingly, a pulse of phosphorylated AKT at Ser473, which is coincident with the leading edge of barrier formatting skin is increased in spinous- and granular layers at E16.6∼18.5 (Ding et al., 2016; O'Shaughnessy et al., 2007; O'Shaughnessy et al., 2009), indicating that mTORC2 activity is transiently activated during epidermal barrier formation. Indeed, we and others showed that epidermal-specific deletion of *Rictor* results in reduced phosphorylation of AKT-S473 in the differentiated upper layers. The epidermis is thin and hypoplastic, but the barrier function is less severely compromised compared with mTORC1-deficient epidermis (Ding et al., 2016; Tassone et al., 2017). Taken together with results from immunohistochemical staining, it can be concluded that both mTORC1 and mTORC2 are spatiotemporally activated and regulate epidermal barrier formation in a cooperative and nonredundant manner during development. Each complex exerts different functions during epidermal stratification. mTORC1 plays a large role in epidermal differentiation and tissue growth, whereas mTORC2 may control epidermal stratification, terminal differentiation and possibly other processes.

Aside from the above mouse models, studies with mTOR signaling associated molecules further emphasized the critical involvement of mTOR activity in the epidermal barrier formation. Mice with epidermal-specific deletion of IGF-1 receptor and 3-Phosphoinositide-dependent protein kinase-1 (PDK1), the major upstream controller of mTOR signaling, exhibit an impaired skin barrier function with varied severity (Stachelscheid et al., 2008; Gunschmann et al., 2013; Dainichi et al., 2016). mTORC1 activation requires its translocation from the cytosol to the lysosomal surface (Condon and Sabatini, 2019). Intriguingly, a recent study using organotypic human skin demonstrates that lysosomal activity-mediated mTOR signaling activation is required for human epidermal differentiation (Monteleon et al., 2018), indicating a conserved role of mTORC1 signaling activation in both human and mouse epidermal differentiation. Moreover, activated mTOR signaling suppresses autophagy. Aligned with this notion and the hyperactivated mTOR signaling during epidermal development, autophagy likely plays a limited role for epidermal barrier formation, as mice with epidermal-specific deletion of autophagic genes *Atg5* or *Atg7* do not display any obvious barrier defects (Rossiter et al., 2013; Sukseree et al., 2013).

By contrast, in the adult skin, phosphorylated- S6 and AKT at Ser473 are predominantly detected in the granular layers of the IFE (Squarize et al., 2010; Ding et al., 2020), suggesting that the activation of mTOR is restricted in terminally differentiating cells of the adult IFE. Although cells at granular layer are differentiated and non-proliferative, they are likely active in metabolism and actively synthesize keratohyalin granules, containing the barrier proteins filaggrin and loricrin, lipid-enriched lamellar bodies, as well as cytokines, and antimicrobial peptides (AMPs) (Jiang et al., 2020). However, the mechanism and functional role of the specific-activated mTOR signaling in the granular layer, which could be crucial for maintaining the physiological function of epidermis, remain to be investigated.

## Dynamic mechanistic target of rapamycin signaling activation controls hair growth

**FIGURE 3:**
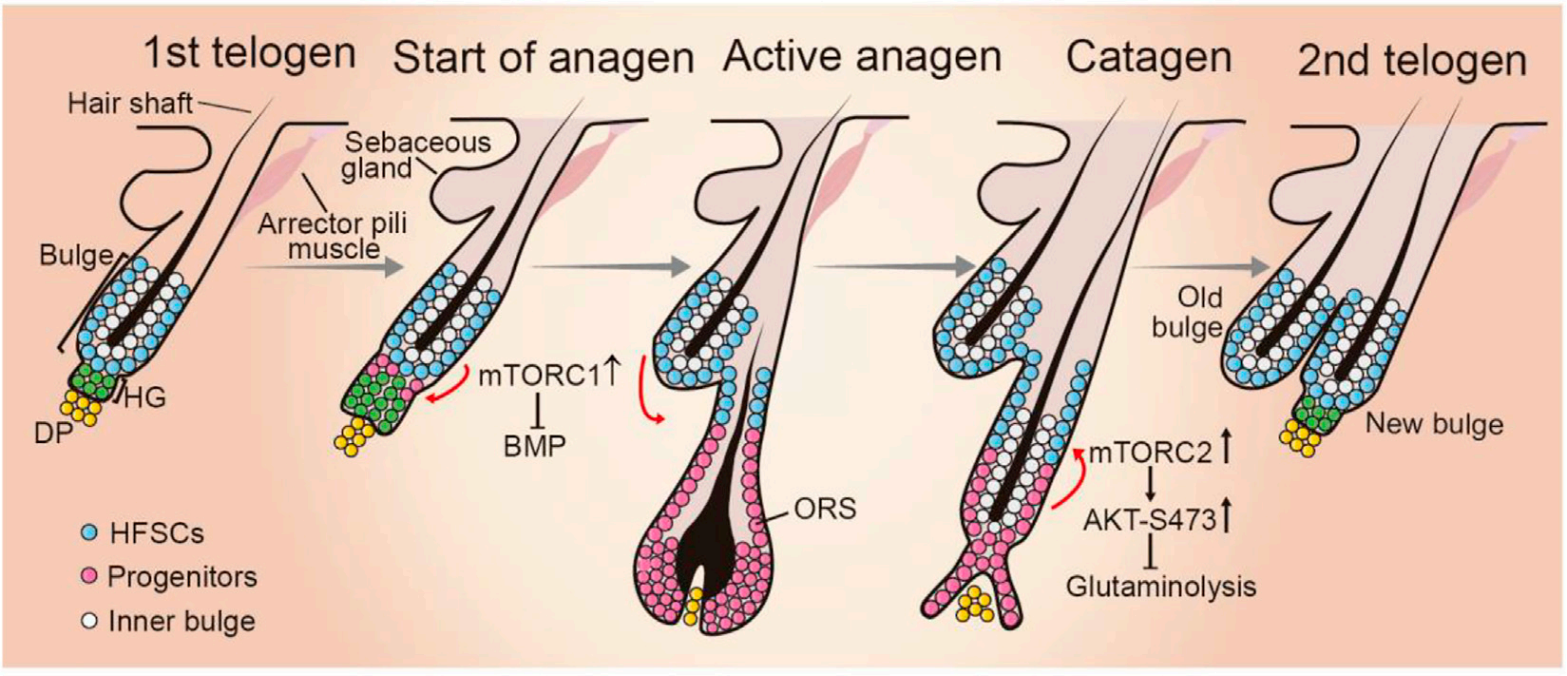
Role of mTOR signaling during hair follicle cycling. HF cycling progresses with resting phase (telogen), growth phase (anagen), and regression phase (catagen). At telogen, HFSCs reside in the out layer of bulge in quiescence. In response to environmental cues, hair germ (HG) cells proliferate and initiate anagen phase. HFSCs start proliferate. The progeny migrates downward and forms the structures of new hair follicle. When HF cycling enters catagen phase, the bulge HFSC niche is re-established and become quiescent. mTORC1 activity is critical for HFSC differentiation by suppressing BMP signaling when HF cycling enters anagen phase, whereas mTORC2 plays a role in establishing HFSC niche of newly formed HF at the anagen-catagen transition period by suppressing glutaminolysis. DP, dermal papilla; ORS, outer root sheet.

HF in postnatal skin undergoes growth cycles, including phases of growth (anagen), regression (catagen), and rest (telogen) (Figure 3). Hair follicle stem cells (HFSCs), residing in the bulge area, govern the transition through these phases (Hsu et al., 2014). HFSCs change from a dormant to an active state and return quiescence in response to cues from their microenvironment. Bone morphogenetic protein (BMP) pathway is necessary for HFSC quiescence, whereas the Wnt pathway is critical for HFSC activation and hair cycle initiation. The mechanisms regulating HFSCs and HF cycling have been nicely summarized in the recent review articles (Yi, 2017; Li and Tumbar, 2021). Although the primary molecular signals that control HFSC self-renewal, and the transition between quiescence and activation have been recently identified, how these signals synergistically to regulate hair cycles remains elusive.

Accumulating evidence suggest mTOR signaling is critically involved in HF cycling in many aspects. Intriguingly, mTORC1 activation exhibits phase-dependent changes and phosphorylated mTOR is detected in certain sites of HFs in a phase-dependent manner (Kellenberger and Tauchi, 2013). mTORC1 signaling is activated in HFSCs at the time point of telogen-to-anagen transition. Rapamycin treatment or genetic inhibition of mTORC1 significantly delays HFSC activation and extends the telogen period (Kellenberger and Tauchi, 2013; Deng et al., 2015). Mechanistically, mTOR signaling likely promotes HF cycling and HFSC activation by suppressing BMP signaling during hair regeneration (Deng et al., 2015).

Along with these observations, several lines of evidence indicate that HFSC pool maintenance and HF cycling are influenced by the altered mTORC1 signaling activity. Calory restriction (CR) can expand lifespan and retard the onset of age-related disease and at the molecular level these effects appear to be largely overlapping mTORC1 inhibition (Lopez-Otin et al., 2013; Saxton and Sabatini, 2017; Madeo et al., 2019). Intriguingly, systemic CR in mice can enlarge the HFSC pool, promote hair follicle growth and retention rates (Forni et al., 2017). Consistently, with a context of cultured human oral keratinocytes, inhibiting mTORC1 by rapamycin treatment facilities stemness maintenance (Iglesias-Bartolome et al., 2012). Conversely, mTORC1 signaling is activated after irradiation and it is required for timely regeneration of the hair follicles and hair growth after radiation injury (Wang et al., 2017). However, sustained activation of mTORC1, triggered by constitutive activation of Wnt signaling leads to HFSC senescence and consequently stem cell pool exhaustion (Castilho et al., 2009), indicating that persistent mTORC1 activation during repeated regeneration impairs somatic stem cell maintenance. Similar observation has been also reported in hematopoietic and muscle stem cells (Haller et al., 2017). Taken together, mTORC1 activation is required for the exit from quiescence of HFSC and mTORC1 thus may act as a gatekeeper through the molecular cascades that regulates HF cycle initiation.

Compared to mTORC1, the role of mTORC2 in hair growth remains less investigated. Mice expressing an active form of AKT in the epidermis exhibit hair follicle hyperplasia (Segrelles et al., 2007), but the underlying molecular mechanism remains unclear. During mouse hair follicle cycling, phosphorylated AKT at Ser473 is predominantly detected in the upper outer root sheet (ORS) of anagen-catagen transition phase (Alonso et al., 2005; Kim et al., 2020), indicating that mTORC2 is activated in the ORS progenitor cell population. Combining *in vitro* and *in vivo* experiments, we found that mTORC2-mediated AKT activation suppressing glutaminase expression and glutamine metabolism is essential for progenitor fate reversibility and HFSC niche establishment during anagen-to-catagen transition period (Kim et al., 2020). Consequently, epidermal-specific *Rictor* deficient mice display a failure to re-establish the HFSC niche, defected second hair follicle bulge regeneration, and compromised long-term maintenance of HFSCs and hair loss in aged mice (Kim et al., 2020). These findings highlight a critical role for the temporally activation of mTORC2-AKT signaling axis in regulating HFSC homeostasis and HF cycling progression.

Collectively, mTOR signaling is critically involved in hair follicle growth at multiple phases by influencing HFSC fate. Of interest, mTORC1 and mTORC2 appear apposing functions in regulating HF cycling and HFSC pool maintenance, where mTORC1 activation is crucial for HFSC differentiation, thereby modulating the timing of the onset of anagen, while activated mTORC2 facilitates the switch ORS progenitors back to the quiescent HFSC state, establishment new bulge niche after the catagen (Figure 3). These observations highlight the critical importance of spatiotemporal mTOR signaling activation during hair cycle. Further unveiling the mechanisms that control mTOR signaling activation during hair growth may provide new approaches to maintain stable HFSC pool and their regenerative capacities.

## Activated mechanistic target of rapamycin signaling drives epithelization during skin repair

Wound healing is a dynamic and complicated process that occurs in sequential phases: inflammation, proliferation, and remodeling, involving diverse type of cells, growth factors and cytokines, and extracellular matrix (Gurtner et al., 2008). Immune cells such as neutrophils, monocytes, and macrophages are recruited at the wound bed to clear bacteria, debris, and foreign materials (Eming et al., 2007). Wound edge keratinocytes are activated by a variety of chemokines and cytokines, along with mechanical and physical microenvironmental alterations, and exhibit enhanced migration and proliferation activities, forming a hyperproliferative epithelium (HE) tongue. The efficient proliferation and migration of wound HE keratinocyte plays a vital role in restoration of the injured skin. Compromised epithelialization often results in skin ulcers and chronic wounds (Pastar et al., 2014). Studies with living microscopy have demonstrated that a proliferating ring grows around the injured area but away from the wound edge. In the proliferative ring, cell division is stimulated and originated toward the wound inside to rebuild the lost epithelial cells. Simultaneously, cells migrate towards the wound bed by rearranging, flattening and elongating their shapes (Aragona et al., 2017; Park et al., 2017). Independent lines of evidence suggest that the mTOR signaling activity is an important regulator of keratinocyte motility, proliferation, and differentiation *in vitro*. Laser phototherapy or hypoxia stress promotes human keratinocytes migration and accelerates wound closure *via* inducing mTOR activity (Pellicioli et al., 2014; Yan et al., 2017). Conversely, keratinocytes with rapamycin treatment or mTORC2 deficiency display reduced proliferation rates (Javier et al., 1997; Tassone et al., 2017).

**FIGURE 4:**
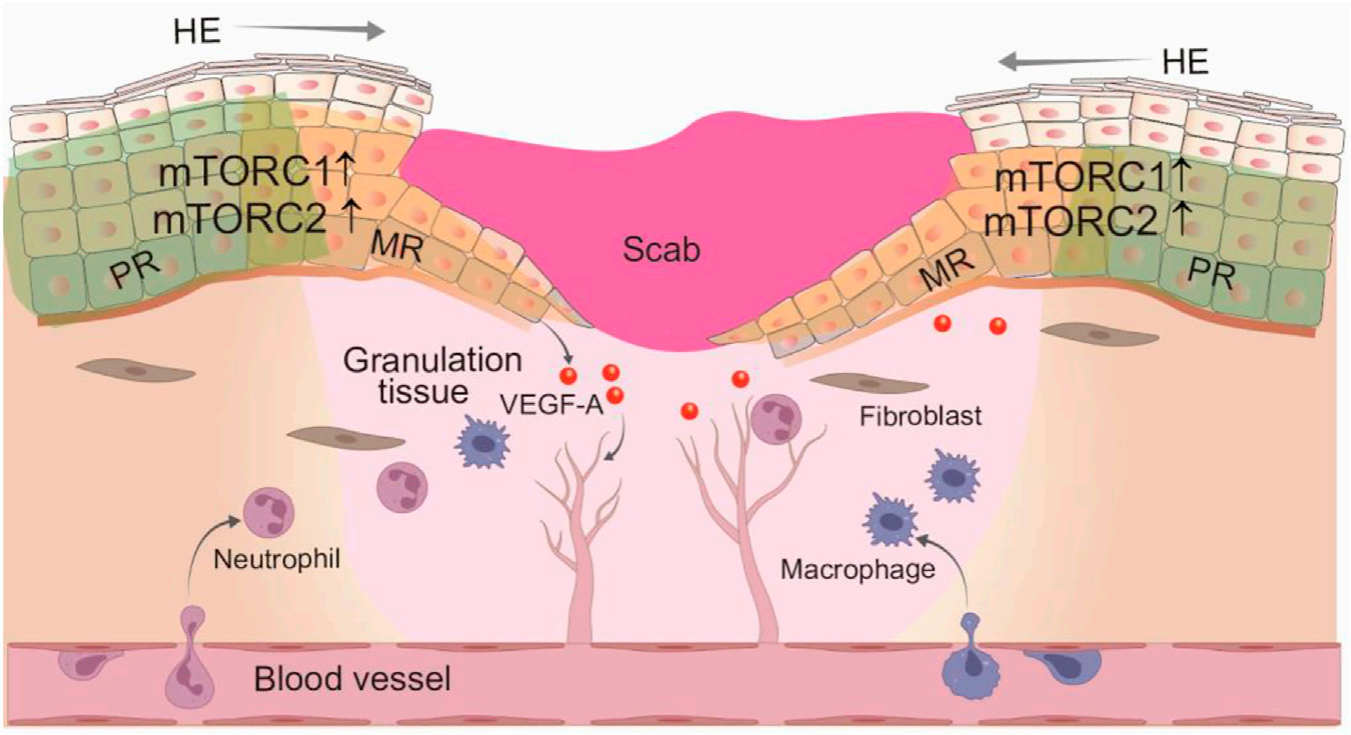
Regulation of wound epithelization and angiogenesis by mTOR signaling. Skin wound healing is a well-organized process that involves dynamic and overlapping phases: inflammation, cell proliferation and migration, and tissue remodeling. In response to various growth factors and cytokines, multiple sourced keratinocytes at the wound edge are activated to promote epithelization through enhanced proliferation and migration. Both mTORC1 and mTORC2 signaling activity are robustly induced in the hyperproliferative wound epithelium, promoting wound cell proliferation and migration. In addition, activated mTORC1 also regulates angiogenesis through increased VEGF-A expression. HE, hyperproliferative epithelium; PR, proliferative ring; MR, migration ring.

Given the similarities between embryonic tissue development and wound repair (Park, 2021), the role of mTOR signaling in epidermal morphogenesis also translates into skin repair. The direct hint of an important role of mTOR in skin wound healing is the robust activation of mTORC1 (pS6) and mTORC2 (AKT-pS473) in all layers in the keratinocytes of migration ring, meanwhile with an activation of mTOR in spinous layer in the keratinocytes of proliferation ring, following wound healing (Squarize et al., 2010) (Figure 4). The injury-induced mTOR activation in epithelial cells is likely conserved among species, as it is also observed in *Drosophila melanogaster* larvae epithelial repair and zebrafish retinal pigment epithelium regeneration (Kakanj et al., 2016; Lu et al., 2022). Clinic studies have revealed similar importance for mTOR in skin wound healing, as evidenced by the development of skin ulcerations and chronic wounds following treatment with mTOR inhibitor, everolimus, to prevent graft rejection in graft-versus-host-disease (Law, 2005; Feldmeyer et al., 2012; Brown et al., 2014). This side effect is reversible after withdrawal of rapamycin application (Brown et al., 2014). Besides, several genetic studies in mice further support that mTOR signaling activity is critical for wound healing efficiency *in vivo*. Epidermal-specific deletion of *Pten*, *TSC1*, and *AMPK*, the well-documented negative regulators of mTOR signaling leads to the increased phosphorylated S6, AKT (Ser473) at the wound HE tongues, enhanced wound keratinocyte proliferation and migration, and the accelerated wound epithelization (Squarize et al., 2010; Crane et al., 2021). Altogether, the activation of mTOR signal is essential for efficient wound healing, presenting the possibility that inducing mTOR action may be a promising treatment for wound healing.

Chronic wounds often occur in patients with diabetes mellitus (DM) and represent major clinical and socio-economic problems (Eming et al., 2014; Nunan et al., 2014). Although the molecular mechanisms underlying the compromised wound healing capacity of DM are complicated, recent findings suggest that the impaired wound healing has been associated with failed activation of the mTOR pathway in the epidermis of diabetic skin (Jere et al., 2019). The activation of mTOR signaling pathway is remarkably attenuated in the wounds of diabatic mouse and rat, compared to non-diabetic wounds (Goren et al., 2009; Huang et al., 2015). One of the key characteristics of chronic wounds is attenuated angiogenesis, which is regulated by VEGF-A (Galiano et al., 2004). It has been shown that epidermal mTOR signaling functions as contributors of VEGF-A expression, thereby affecting chronic wound healing. AKT-1-mediated VEGF-A production from wound keratinocytes largely dependent on mTORC1-4E-BP1 signaling axis activity in normal skin wound. Notably, p-4E-BP1 level is significantly decreased in diabetic wound keratinocytes, which correlates with reduced VEGF-A expression (Goren et al., 2009). These data demonstrate a role of epidermal mTOR in regulating angiogenesis during wound healing, highlighting the importance of cellular cross-talk during tissue repair. Taken together, since the increased mTOR signaling at the wound edge is positively correlated with wound epithelization, the deficiency of mTOR signlaing activity may represent one of the key factors preventing wound closure and eventual disturbed repair in diabetes mellitus. Therefore, targeting on mTOR signaling pathway may be a viable approach to promote wound healing processes.

## Mechanistic target of rapamycin signaling in skin diseases

Dysregulated mTOR signaling is tightly associated with the development of several hyperproliferative skin disorders, including skin cancer, and inflammatory skin diseases (Buerger et al., 2013; Mossmann et al., 2018; Varshney and Saini, 2018; Deng et al., 2021). Because of the width of this topic, in this section we mainly discuss the implications of mTOR signaling pathway in keratinocyte carcinomas (also known as non-melanoma skin cancers, NMSC), in particular basal cell carcinoma (BCC) and squamous cell carcinoma (SCC), and inflammatory skin diseases, such as psoriasis and AD. The causal links between mTOR signaling activities and skin disorders are summarized in Table 1.

**TABLE 1:** Dysregulated mTOR signaling in various skin disorders.

Skin disorder	Altered activity/biological affection	Reference
BCC	p-mTOR, p-S6 and p-AKT levels are increased in a subpopulation of BCC patients/cell proliferation, carcinogenesis initiation and development	Karayannopoulou et al. (2013); Brinkhuizen et al. (2014)
SCC	p-mTOR, p-S6K, p-4E-BP1, and p-AKT levels are significantly increased in human SCC biopsies/carcinogenesis initiation and development	Chen et al. (2009b)
PD	Levels of p-S6K and p-S6 are increased in PD mouse model skin; p-S6 is increased in EMPD patient skin/contribution to Paget-like phenotype and PD development	Chen et al. (2009a); Song et al. (2020)
Psoriasis	p-mTOR is increased in basal layer; p-S6K, p-S6 are increased in suprabasal layers of lesional psoriatic skin/cytokine production, cell proliferation	Buerger et al. (2013); Burger et al. (2017)
AD	RAPTOR expression inversely correlates filaggrin level in AD skin; mTORC2-AKT plays a role in filaggrin processing/skin barrier function	Naeem et al. (2017); Osada-Oka et al. (2018); Ding et al. (2020)
Rosacea	mTORC1 activity is elevated in human skin biopsies and mouse model skin/inflammation; LL37 expression	Deng et al. (2021)

### Mechanistic target of rapamycin and skin cancer

BCC is the most common form of human NMSC, which primarily arise from epidermal basal layer keratinocytes and is often provoked by ultraviolet (UV) radiation exposure, as well as trauma and injury (Peterson et al., 2015). Compared to BCC, SCC is less frequent and mostly originates from squamous cells of epidermis, but it is more aggressive and dangerous (Madan et al., 2010). Compared to normal skin, skin tumor tissues contain significantly elevated levels of phosphorylated mTOR and the downstream effectors S6K, 4E-BP1, and AKT (Ser473), which are implicated in increased cell proliferation, tumorigenesis, and resistance to apoptosis (Chen S. J. et al., 2009b). Intriguingly, compared to BCC, SCC displays a considerably higher level of phosphorylation of mTOR, thereby exhibiting high sensitivity to mTOR inhibitors (Karayannopoulou et al., 2013; Brinkhuizen et al., 2014).

Genetic studies in mice further consolidate the implication of mTOR signaling activity in skin tumorigenesis. Loss of *Pten*, a key negative regulator of mTOR, leads to an SCC tumorigenesis, which at least in part due to activated mTORC1, since *Raptor* deletion in epidermis suppress tumorigenesis in *Pten* mutants (Hertzler-Schaefer et al., 2014). Conversely, mouse models with ectopic overexpression of PRAS40, an inhibitory component of mTORC1, suppresses 7, 12-dimethylbenzanthracene (DMBA)/12-O-tetradecanoylphorbol-13-acetate (TPA)-induced skin tumor development (Rho et al., 2016). Conditional disruption mTORC2 in epidermis by inducible deletion *Rictor* is sufficient to delay tumor development and trigger regression of established tumors (Carr et al., 2015), indicating that mTORC2 activation is essential for SCC development and inhibition of mTORC2 may be valuable in tumor therapy. Human papillomaviruses (HPV) are classified as another skin carcinogenic factor and their infections can lead to develop SCC later in life (Ding et al., 2015). mTOR signaling activity, specifically pS6 accumulates in multiple HPV-associated cancers (Molinolo et al., 2012; Madera et al., 2015). mTOR inhibition abolishes SCC initiation and development upon carcinogen exposure of HPV16 transgenic mice (Callejas-Valera et al., 2016). Thus, mTOR signaling pathway represents a potential therapeutic target for preventing HPV-associated cancers.

Extramammary and mammary glands Paget’s diseases (PD) are a kind of rare, but malignant epidermal cancer that is distinguished by the presence of Paget cells. Despite its ease of diagnosis by histological analysis, the molecular mechanism underlying PD pathogenesis remains unclear. Single-cell transcriptomes of Paget cells from human and mouse extramammary PD samples along with functional studies have identified that mTORC1 signaling pathway is activated and inhibition of mTOR signaling with rapamycin efficiently suppresses the Paget-like phenotype (Chen S. et al., 2009a; Song et al., 2020) (Table 1).

Collectively, these studies demonstrate that aberrant mTOR signaling activity in skin carcinogenesis and raise inhibiting mTOR signaling as an attractive therapeutic target in skin cancer (Chamcheu et al., 2019). Of note, treatment with mTOR inhibitors is more effective in preventing tumors at the initiation and promotion stages. While, for the established SCC, only mTOR inhibition in combined with other therapy, e.g., radiotherapy can yield positive effects in inducing tumor regression (Darido et al., 2018; Patel et al., 2020), highlighting the temporal regulation of mTOR on tumor development and the importance of time window for the administration.

### Mechanistic target of rapamycin and inflammatory skin diseases

Psoriasis is a chronic inflammatory skin disease that is characterized by red, scaly plaques and epidermal thickening. The proinflammatory cytokines, generated by both immune cells and keratinocytes, such as TNF-α, IL-17A and IL-23, induce and aggravate the development of psoriasis (Hawkes et al., 2017). Several lines of evidence have demonstrated that abnormal mTOR signaling activation are associated with psoriasis. Hyperactivated mTOR signaling, including phosphorylated- S6K-1, 4E-BP1 and AKT, are observed in lesional skin of psoriasis patients, supporting a pathological function of mTOR in psoriasis (Madonna et al., 2012; Buerger et al., 2013; Buerger et al., 2017; Buerger, 2018) (Table 1). Inflammatory cytokines, including IL-22, IL-17, TNF-α can induce the aberrant activation of mTOR cascade in keratinocytes, leading to enhanced keratinocyte proliferation and reduced differentiation, which are the hallmarks of psoriasis (Mitra et al., 2012; Buerger et al., 2017). Concomitantly, mTOR signaling pathway in turn enhances keratinocytes to produce proinflammatory molecules, including IL-6, CXCL8 and VEGF in response to TNF-α, suggesting that a feedback loop driven by mTOR signaling triggers and aggravates the pathogenesis of psoriasis. Besides, appropriate autophagy is required for epidermal function (Akinduro et al., 2016). A recent report demonstrates that mTORC1 might also promote pathogenesis of psoriasis by its inhibition on autophagy (Varshney and Saini, 2018). In mice, topical activating mTORC1 activity on the skin with selective mTORC1 agonist recapitulates psoriasis-like phenotype in mouse skin (Buerger et al., 2017). Topical or systemic administration of mTOR inhibitor potentially ameliorates the psoriatic phenotype (Ormerod et al., 2005; Burger et al., 2017; Gao and Si, 2018). Collectively, the altered mTOR signaling is critically involved in psoriatic pathogenesis and targeting on mTOR signaling may represent an innovative therapeutic approach in psoriatic treatment.

AD is considered as the most common chronic immunoinflammatory skin disease that presents with impaired barrier function and enhanced percutaneous activity to environmental stimuli (Ständer, 2021). Aberrant activation of mTOR signaling seems also implicated in the development of AD. AD induced by DNFB in mice shows increased activation of mTORC1 signaling (Osada-Oka et al., 2018) (Table 1). Importantly, pharmacological inhibition of mTORC1 in an AD mouse model induced by DNFB relieves several clinical symptoms, such as inflammatory cell infiltration, reduction in clinical skin condition score and serum IgE levels (Yang et al., 2014; Jung et al., 2015). Intriguingly, elevated RAPTOR expressions have been observed in patients with AD. The effect of increased RAPTOR expression seems to correlate with strong inhibition of filaggrin procession in the epidermis through inhibiting AKT activity, highlighting the importance of the crosstalk between mTORC1 and mTORC2 in the context of AD (Naeem et al., 2017). In line with this, we recently demonstrated that mTORC2-mediated AKT activity is essential to filaggrin processing. Mice with *Rictor* deletion in the epidermis exhibit an ichthyosis-like phenotype with altered skin barrier function and enhanced percutaneous immune responses (Ding et al., 2020). While the precise mechanisms by which how mTORC1 and mTORC2 cooperate in controlling the pathogenesis of AD is currently under investigation, selectively inhibiting mTORC1 signaling pathway may represent an attractive target in AD therapy.

Rosacea is another chronic skin inflammatory disease that is characterized by erythema, papules, pustules and chronic edema (Buddenkotte and Steinhoff, 2018). The role of mTOR is less investigated in rosacea than in AD and psoriasis. Studies with RNA-sequencing of lesioned skin from rosacea patients identify mTOR signaling as a critical pathway in the pathogenesis of rosacea (Deng et al., 2021). In the context of the LL37-induced rosacea-like mouse model, mTORC1 signaling, but not mTORC2, is activated in epithelial cells (Table 1). Consistently, ablation of *Raptor* in epidermis suppresses rosacea development in mice. Meanwhile, in the reciprocal condition, heterozygous deletion of TSC2, which negatively regulate mTORC1, promotes and aggravates rosacea development. Mechanistically, cathelicidin LL37, a hallmark of rosacea, activates mTORC1 signaling activity, which in turn not only induces the production of LL37 but also NF-κB activation and disease-associated cytokines in keratinocytes. Moreover, mTORC1-mediated angiogenesis is also implicated in the development of rosacea (Peng et al., 2021). Remarkably, patients with topical application of mTORC1 inhibitor showed relieved rosacea symptoms (Deng et al., 2021). Taken together, these findings strongly demonstrate the altered epidermal mTOR signaling status under skin inflammatory disease conditions and suggest that inhibition of mTOR signaling activity as a promising treatment for inflammatory skin disease in general (Karagianni et al., 2022).

### Mechanistic target of rapamycin and viral skin infection

Viral infection affects skin function in various aspects and virus replication requires cellular ribosomes and energy metabolism to translate their proteins and compose other components. Increasing evidence shows that viral skin infection is tightly associated with mTOR activity. As mentioned above, continuous HPV infection may trigger skin tumorigenesis, such as cervical and oropharyngeal cancer. mTOR signaling is activated in human keratinocytes upon exposure to HPV16, as observed by increased phosphorylation of 4E-BP1, S6K and AKT on Thr308 and Ser473 (Surviladze et al., 2013). Consequently, mTOR inhibition significantly reduced HPV16 infection *in vitro*. This effect seems to be associated with mTOR-mediated autophagy (Surviladze et al., 2013). Coronavirus disease 2019 (COVID-19) is a severe pandemic infectious disease all over the world since 2019 and is caused by a coronavirus, named severe acute respiratory syndrome coronavirus 2 (SARS-CoV-2). In addition to the typical respiratory symptoms, including fever and pneumonia, several symptoms reported cutaneous manifestations, including erythematous rash, widespread urticaria, and chickenpox-like vesicle (Jin et al., 2020; Recalcati, 2020). Single-cell sequencing and immunohistochemistry analyses demonstrate that angiotensin converting enzyme 2 (ACE2), a main functional receptor of SARS-CoV-2, is highly expressed by human keratinocytes, indicating the potential routes of percutaneous infection (Xue et al., 2021). Furthermore, it has been shown that COVID-19 infected patient tissues and epithelial cells exhibit the abnormally activated mTOR signaling pathway, as evidenced by increased levels of phosphorylated 4E-BP1, AKT, S6K1 and mTOR (Appelberg et al., 2020; Mullen et al., 2021), suggesting the relevance of mTOR signaling activity and SARS-CoV-2 infection. More importantly, small molecule inhibitors targeting AKT and mTOR and engineered extracellular vesicles encapsulated microRNA targeting mTOR are able to significantly antagonize SARS-CoV-2 infection (Ibrahim et al., 2022), highlighting that inhibition of mTOR represents a feasible strategy for preventing COVID-19 (Basile et al., 2022).

## Conclusion

Here, we reviewed recent understanding of epidermal mTOR signaling in the context of skin health and disease. The ongoing evidence underscores that an appropriate and dynamic mTOR signaling activation is necessary for skin barrier formation, homeostasis, and inflammation response by acting as a regulator of keratinocyte differentiation, proliferation, and cytokine and growth factor production. Dysregulated mTOR signaling pathway can cause skin barrier defect, delayed wound healing, tumorigenesis, and inflammatory diseases. Therefore, modulating mTOR signaling may provide potential therapeutic opportunities for skin disorders. However, despite increasing evidence of the involvement of mTOR signaling pathway in the physiopathological of skin homeostasis, the precise mechanisms by which mTOR regulates skin homeostasis and the development of skin diseases are complicated and far from entirely understood. The spatiotemporal activation of mTOR signaling may be triggered by the diverse combinations of environmental cues, including nutrients, growth factors/cytokines, and calcium (Li et al., 2016). It is largely unknown which upstream inputs induce mTOR signaling during skin homeostasis and its dysregulation in pathological conditions. Identifying the specific upstream regulators may provide an opportunity to enable the cell to an appropriate biological response through mTOR signal modulation. Furthermore, it is equally important to identify the functional role of downstream effectors of mTOR signaling activation. Studies in *D. melanogaster* larvae epithelium have revealed that S6K is the major downstream effector of mTOR signaling during the larvae epithelium wound healing (Kakanj et al., 2016). Moreover, identifying crucial targets of mTOR signaling, e.g., at translation level will be a significant step forward for the possible therapeutic application in the future.
